# Preference and In Vitro Digestibility of Leaves of Woody Plants by Sheep in the Northern Sudanian Zone

**DOI:** 10.1111/jpn.70032

**Published:** 2025-11-28

**Authors:** Linda C. Gabriella Traore, Sita Sanou, H. Oumou Sanon, Regina Roessler, Valérie Bougouma‐Yameogo, Eva Schlecht

**Affiliations:** ^1^ Ecole Doctorale Sciences Naturelles et Agronomie (SNA) Université de Nazi Boni Bobo‐Dioulasso Burkina Faso; ^2^ Département de Production Animales Institute de l'Environnement et de Recherche Agricoles du (INERA) Ouagadougou Burkina Faso; ^3^ Animal Husbandry in the Tropics and Subtropics University of Kassel and University of Göttingen Witzenhausen Germany; ^4^ Institut du Développement Rural (IDR) Université Nazi Boni Bobo Dioulasso Burkina Faso

**Keywords:** browse fodder, cafeteria test, condensed tannins, Hohenheim gas test, sheep

## Abstract

In West Africa, trees and shrubs are important for feeding ruminant livestock during the dry season. This study aimed to determine the in vitro digestibility of organic matter from eight woody species using a gas test with and without the addition of polyethylene glycol (PEG), and evaluate their preference by sheep using a cafeteria test. Plants cited by farmers as being palatable to sheep were *Lannea microcarpa* (La), *Ficus sycomorus* (Fi), *Pterocarpus erinaceus* (Pt), *Khaya senegalensis* (Kh), *Azadirachta indica* (Az), *Bombax costatum* (Bo), *Guiera senegalensis* (Gu) and *Ziziphus mauritiana* (Zi). For the preference test, two groups of fresh and dried leaves from each time four species were offered in a 4 × 4 Latin square to four 18–24‐month‐old rams for 8 days each. The in vitro organic matter digestibility (IVOMD) was determined using the modified Hohenheim gas test. The quantity of dry matter ingested within 30 min, along with consumption time, ingestion rate, and the preference coefficient, served as indicators of leaf preference. Bo, Kh, and Zi leaves had the highest preference coefficients both in the dried (0.7, 0.3 and 0.2) and fresh (0.7, 0.5 and 0.3) state. Fresh Fi leaves had a higher preference coefficient (0.4) than dried ones (0.1), while the reverse was observed for Pt leaves (fresh: 0.1, dried: 0.6). PEG addition increased IVODM and in vitro methane production of ligneous forage plants by 1.2% (Kh) to 44.7% (La) compared to the incubation without PEG. In conclusion, fresh and dried leaves of *B. costatum, K. senegalensis*, and *Z. mauritiana* are highly palatable to sheep, making them good candidates for inclusion in dry season rations, despite their moderate IVOMD. In contrast, *L. microcarpa* and *G. senegalensis* exhibit both low preference and poor IVOMD, rendering them less recommendable as forage resources.

## 1 Introduction

Trees and shrubs are an essential part of ruminant diets in sub‐Saharan Africa, especially in Burkina Faso, where livestock farming primarily relies on natural vegetation (Traore et al. 2011; Sarr et al. 2013). The long dry season of 7–9 months considerably reduces the availability of herbaceous biomass to a point of near absence (Diatta et al. 2023). Feed scarcity is aggravated by a depletion of crop residues due to harvest and homestead feeding, or intense stubble grazing, and expensive agro‐industrial by‐products (Silue et al. 2014; Dione et al. 2020). In this situation, livestock farmers increasingly rely on diverse and protein‐rich woody fodder species (Dossou et al. 2012; Traore et al. 2023). Thereby, it is advantageous that several woody species like *Piliostigma reticulatum, Faidherbia albida*, *Acacia nilotica*, *Balanites aegyptiaca*, *Khaya senegalensis*, *Pterocarpus erinaceus*, and *Ficus sycomorus* maintain their leaves during the dry season (Mahamane et al. 2007; Arbonnier 2009). However, it is important to note that animals selectively consume woody fodder. Baumont (1996) defined palatability as food characteristics driving an animal's selective response. Provenza (2006) further explained that the animal's preference of forage species depends on several factors including the plant's physical characteristics (height, thorn presence, moisture content, texture) and chemical characteristics (nutrient content and secondary metabolites).

Secondary metabolites are protective compounds produced by plants in response to adverse conditions such as intense sunlight, inferior soil fertility, or pressure from herbivores and microbes (Soulama et al. 2014). As such, these metabolites, including condensed tannins, can act as anti‐nutritional factors. At elevated concentrations, they can impart a bitter taste that reduces palatability and feed intake, and they can also affect the plant's digestibility (Soulama et al. 2014; Nascimento et al. 2021). Given the importance of woody fodder for ruminant feeding during the dry season, identifying species that combine animal preference with good nutritional value, in particular digestibility, is important for advising farmers who opt to stall‐feed their small ruminants. Therefore, the objective of this study was to determine the in vitro digestibility and palatability of woody plant leaves that herders in the northern Sudanian zone of Burkina Faso consider to be preferred by sheep. We thereby hypothesised that the presence of secondary compounds influences both variables, and that an animal's preference varies according to plant species and the fresh or dried state of leaves.

## 2 Materials and Methods

### 2.1 Study Area

The study took place at the Saria Research Station, Regional Directorate for Environmental and Agricultural Research (DRREA/Centre) of the Institute for Environmental and Agricultural Research (INERA). The station is located in the village of Saria, 23 km east of the town of Koudougou and 80 km west of Ouagadougou (latitude 12°16′ N, longitude 2°9′ W). The climate is North Sudanian (Fontès and Guinko 1995). The natural flora predominantly features annual grass savannah. In this area, woody plants are typically situated within agroforestry parks. The latter host 20–49 different woody species, with *Vitellaria paradoxa*, *Lannea microcarpa*, and *Azadirachta indica* being the most prevalent ones (Sehoubo et al. 2023). Other woody species such as *Parkia biglobosa*, *Faidherbia albida*, *Tamarindus indica*, *K. senegalensis*, *Guiera senegalensis*, and *P. reticulatum* are also represented within these parks (Sehoubo et al. 2023).

### 2.2 Selection of Ligneous Forage Plants

The eight forage plants investigated in this study were chosen based on an ethnobotanical survey conducted in June‐July and September 2021 in the study region. A total of 185 agro‐pastoralists were interviewed using a semi‐structured questionnaire to identify woody plants that are most preferred by sheep. Based on the answers (Table 1), the leaves of *F. sycomorus* (Fi), *L. microcarpa* (La), *P. erinaceus* (Pt), *K. senegalensis* (Kh), *A. indica* (Az), *Bombax costatum* (Bo), *G. senegalensis* (Gu), and *Z. mauritiana* (Zi) were identified as candidates for the present study. La, Pt, Bo, and Zi are deciduous species that lose their foliage in the dry season (October to March), while Gu, Fi, and Az are evergreen; leaf shedding and foliation of Kh is continuous throughout the year (Yengué 2020; Abdou et al. 2021; Chimbekujwo 2021). At the time of leaf collection (July, August), Bo, Gu, Kh, and Pt had started leaf development, Zi was flowering, Fi fruiting, and Az and La were in the stage of fruit maturation.

**Table 1:** Woody fodder plants preferred by sheep according to farmers, in decreasing order of frequency of mention (total *n* = 185).

Species	Family	Local name	Frequency (%)
*Ficus sycomorus* L.	*Moraceae*	Kakanga	41.1
*Lannea microcarpa* Engl. & K. Krause	*Anacardiaceae*	Sabga	38.9
*Pterocarpus erinaceus* Poir.	*Fabaceae*	Noinga	33.0
*Khaya senegalensis* (Desr.) A. Juss.	*Meliaceae*	Kuka	22.7
*Azadirachta indica* A. Juss	*Meliaceae*	Neem	21.1
*Bombax costatum* Pellegr. & Vuill.	*Bombaceae*	Voaka	9.7
*Guiera senegalensis* J.F. Gmel.	*Combretaceae*	Wiliwiga	9.2
*Ziziphus mauritiana* Lam.	*Rhamnaceae*	Muguna	6.0

### 2.3 Digestibility of Organic Matter

Fresh leaves from the eight selected woody species were harvested in the village of Saria in September and October 2021, collecting material from a minimum of five randomly selected individuals per species. The drying process involved a 1‐day wilting period in the sun followed by 2–3 days of complete drying in the shade, with the leaves being turned once a day. Approximately 200 g of air‐dried leaf sample material of each harvested individual was then collected into a composite sample per species, roughly ground (2 mm particle size), packed into a zip‐lock plastic bag, labelled and properly stored in a room away from sunlight and moisture until laboratory analysis.

The in vitro organic matter digestibility (IVOMD) of the sampled leaves was determined using the modified Hohenheim gas test (VDLUFA method 25.1; Menke and Steingass 1988; Leberl et al. 2007; VDLUFA 2012). The gas test was carried out from March to May 2022. In total, six syringes, distributed across two incubations, were used to measure total gas and methane (CH_4_) production after 24 h, with polyethylene glycol (PEG) added (Alonso‐Díaz et al. 2009). An additional six syringes were incubated under the same conditions but without PEG added. PEG builds inert complexes with tannins, deactivates them and reduces the negative effect of tannins on the digestibility of the leaves. This process thus facilitates the determination of the biological activity of tannins (Makkar et al. 1995).

After grinding the leaf samples with a ball mill, for each round of incubation three times 200 mg were weighed for the syringes without PEG and three times 250 mg of feed sample and 450 mg of PEG were weighed for the syringes with PEG, inserted in 100 mL glass syringes and stored at 39°C overnight. Before morning feeding, rumen liquor was taken from a fistulated cow fed ad libitum grass hay and 300 g of dairy concentrate feed (Laktaria 18 pell; 18% crude protein, 4% crude fat, 9% crude fibre). Buffered solution of the rumen liquor was prepared under continuous CO_2_ flushing following established protocols. A 30 mL volume of the buffered rumen liquor was filled into each syringe and the initial volume was noted. Three blank syringes, three with standard hay and three with standard concentrate were added to each incubation. Syringes were incubated in a rotary incubator at a constant temperature of 39°C for 24 h. If the production of gas after 8 h exceeded the capacity of the syringe (gas volume reaching about 80 mL), the exact volume of produced gas was noted and the plunger was set back to 30 mL. In this case, the total gas produced after 24 h was obtained by summing the production after 8 h and 24 h. The blank values were subtracted from the gas volume of the samples after 24 h and the net gas volume corrected for the correction factor of the standard hay and concentrate. The total gas production (TGP) after 24 h was expressed in mL/200 mg dry matter (DM).

After 24 h in vitro fermentation, the concentration of CH_4_ in the total gas produced from the leaf samples was analysed by gas chromatography (Thermo Scientific Trace 1300 with autosampler TriPlus RSH). A gas sample was collected using a gas‐tight Exetainer vial and subsequently injected into the gas chromatograph. The CH_4_ concentration was determined by comparing the measured peak with a calibration curve obtained from a standard gas (argon), and CH_4_ production was reported as percentage of uncorrected gas production after 24 h.

In addition, the air‐dry leaves were analysed for their DM, ash, and crude protein (CP) content (nitrogen x 6.25), for which details are given below. TGP, CP, and ash were used to estimate IVOMD using the following formula (Menke and Steingass 1988; Table 13; Equation 43):

$$\begin{array}{rcl} \text{IVOMD} & = & 15.38+0.8453\;*\;\text{TGP}(\text{ml}/200\;\text{mg DM})\\ & & +\;0.0595\;*\;\text{CP}(g/\text{kg DM})+0.0675\;*\;\text{ash}(g/\text{kg DM}). \end{array}$$

### 2.4 Preference Test (Cafeteria Method)

A preference test of leaves of the selected woody plants was conducted in July and August 2022 using the ‘cafeteria’ method. This approach involves simultaneously offering multiple plant species to the animals and observing their feeding behaviour over a period ranging from 15 min (Degen et al. 2010) to 10 h (Kyambu et al. 2021). Following Alonso‐Díaz et al. (2009), our study involved four uncastrated Mossi rams, aged 18–24 months, with an average live weight of 18.3 ± 0.1 kg at the start of the study. They were dewormed with Bolumisol M1 (1/2 tablet per 25 kg) and vaccinated against peste des petits ruminants with Ovivax PPR (1 mL per animal). The rams were housed in individual pens measuring 2.5 m × 1.2 m.

Due to spatial constraints and observational challenges, the leaves of the eight selected woody species were not presented collectively but randomly split into two groups of four species each, structured systematically in a double 4 × 4 Latin square (Appendix 1). The first group consisted of La, Fi, Pt, and Kh, and the second group included Az, Bo, Gu, and Zi. Fresh leaves were harvested in July and August 2022 from at least five randomly selected individuals per species on the outskirts of the village of Saria and distributed the following day. For the test with dried leaves, the drying procedure followed the protocol outlined above.

The preference test lasted 24 days and was subdivided into two 12‐day phases, one phase for fresh and one phase for dried leaves. After a 4‐day adaptation period to the setup, the sheep's feeding behaviour was observed for 8 days in each phase. Group 1 of leaves was fed on the first day and group 2 on the second day, and so on. Per sheep, 650 g of fresh leaves and 200 g of dried leaves, respectively, was weighed each morning using an electronic balance (600 g capacity and 0.1 g accuracy) and offered at 8:00 h in the morning. The feed troughs containing a specific woody species were placed in different positions each day to prevent the rams from developing a preference for a specific position and to give all woody species an equal chance to be selected. In a 4‐day pre‐study where the same positions of the feeders were used to offer hay and concentrate feed, a preference for a certain position by each ram could be excluded. Two observers monitored the feeding behaviour of two rams each for an interval of 30 min. The observers recorded the position of the selected feed trough (i.e., the woody species), and each start time and end time the animal ingested a certain species, from which the consumption time was calculated. The animals' preference for a specific leaf type was evaluated based on four variables:

− Quantity of ingested dry matter (DMI, g DM) per woody species, calculated as the difference between the quantity of leaves offered and the quantity of leaves remaining after 30 min, taking into account the DM content obtained through laboratory analyses of the leaves,
− Consumption time (CONS, min), which is the total time taken to consume the leaves of a species during the 30‐min observation period,
− Ingestion rate (INGEST, g DM/min), which is the quotient of the consumed leaf DM and its total consumption time, and
− Preference coefficient (PC), which is the quotient of the consumed leaf DM of an individual woody species and the cumulated quantity of the consumed leaf DM of all woody plants offered (Kalio et al. 2006). A higher value indicates a higher preference for the leaves of a specific woody plant.

Following the 30‐min observation period, unused leaves were collected from the troughs and weighed. Subsequently, the experimental sheep received 300 g (as fed) of *Pennisetum pedicelatum* straw and 150 g of dried *Faidherbia albida* pods. All feed troughs were removed from the pens at 16:00 h, ensuring a fasting period to stimulate the animals' appetite for the following day. Fresh water was offered ad libitum.

Every morning, samples of the provided feed (200 g of fresh leaves, 50 g of dried leaves) were collected. The fresh leaves were set to dry in open air. This process involved pre‐drying for a day and subsequent drying in the shade while being turned once daily for about 2–3 days until they reached a consistent weight, although duration could vary due to weather conditions. After determination of the air‐dry matter (ADM) content of each sample, all samples were pooled per species for the test with fresh and with dried leaves, respectively. Of each pooled sample, 200 g air‐dry material were stored in zip‐lock plastic bags for later laboratory analysis.

### 2.5 Proximate Composition and Nutritive Value

All leaf samples from the preference test and the gas test were dried in a force draft oven at 50°C for 2 h to remove any moisture taken up during storage and transport. Then, samples were ground to pass a 1 mm mesh screen and dried at 105°C overnight to determine DM (VDLUFA 2012; method 3.1). Subsequently, the same samples were incinerated in a muffle furnace at 550°C for 5.5 h (method 8.1). The ash was weighed, and organic matter (OM) was calculated from the ash content.

The nitrogen (N) content was determined by the Kjeldahl method (method 4.1.1) using a Vapodest Vap 50 s device (C. Gerhardt GmbH & Co. KG, Königswinter, Germany). Two measurements were taken from each sample and the average was calculated. The CP content was calculated by multiplying N by the factor 6.25 (Close and Menke 1986). Based on Van Soest et al. (1991), neutral detergent fibre (NDF, method 6.5.1) and acid detergent fibre (ADF, method 6.5.2), including the residual ash, were determined using a semi‐automatic Ankom^200^ fibre analyser (ANKOM Technology, Macedon, NY, USA). Alpha‐amylase and sodium sulphite were used as reagents for the determination of amylase‐treated NDF (aNDF) and sulphuric acid for the determination of ADF. The concentration of total extractable phenols (TEP) was determined using the Folin‐Ciocalteu method (Makkar et al. 1993). The dried and ground plant sample was extracted with 70% acetone water. The extract was mixed with the Folin‐Ciocalteu reagent, followed by the addition of a 20% sodium carbonate (Na_2_CO_3_) solution. The mixture was incubated at room temperature for 40–60 min, and the absorbance measured at 725 nm using a Specord 50 Plus spectrophotometer (Analytik Jena, Germany). For the extraction of condensed tannins (CT), the butanol‐HCl method was used (Porter et al. 1986). Dried and ground plant samples were extracted using 70% acetone water. For analysis, the supernatant was mixed in a vortex mixer with a solution of butanol, hydrochloric acid, and ferric ammonium sulphate as a catalyst. The mixture was then heated in a water bath at 97°C for 30 min. After that, the absorbance was measured by spectrophotometry at 550 nm. TEP were expressed as tannic acid equivalents, and CT as leucocyanidin equivalents.

### 2.6 Statistical Analysis

Data were entered into Microsoft Excel 2019 and analysed using R version 4.1.3 (R Core team, 2022). Extreme values were identified and removed using boxplots.stats()$out from the ggpubr package (Kassambara 2022). The normality of the variables was verified using the Shapiro‐Wilk test of the rstatix package (Kassambara 2023). Statistical significance was declared at the 5% level.

For the statistical analysis of the Hohenheim gas test data, the means of IVOMD, TGP, and CH_4_ were calculated from three syringes per sample in each run, separately for samples incubated with and without PEG. Simple linear models were applied to assess statistical differences between plant species, followed by the Tukey HSD post‐hoc test for pairwise comparison of plant species. Residuals were checked graphically with ggqqplot using the ggpubr package (Kassambara 2022), while the homogeneity of variances was evaluated with the Bartlett test using the rstatix package (Kassambara 2023). Pearson correlations were calculated with the tidyverse package (Wickham et al. 2019) between secondary plant compounds (TEP, CT), and IVOMD, TGP, and CH_4_, as well as between PC and IVOMD using the results from incubations without PEG.

The data of the preference test were analysed by group (group 1, group 2) and by state of the leaves (fresh, dried). The dependent variables were DMI, CONS, INGEST, and PC. Leaf type and position of the trough were the independent variables. Generalised linear models were used for DMI and CONS. In case of significant differences, Kruskal‐Wallis test or Wilcoxon test of the rstatix package were used for pairwise comparison (Kassambara 2023). Using the tidyverse package (Wickham et al. 2019), a Kendall correlation analysis was conducted to examine the relationship between DMI and the concentration of proximate components as well as TEP and CT.

## 3 Results

### 3.1 Nutritional Profile of Leaves of the Selected Woody Plants

The proximate composition and concentration of secondary plant compounds (all in DM) of the leaves used in the Hohenheim gas test and in the preference test are presented in Tables 2 and 3, respectively. The variability of the sample means is presented in brackets as the standard error of the mean (SEM). CP concentration of the leaves used in the Hohenheim gas test ranged from 8% (Fi) to 17% (Bo), with an average of 13% (SEM: 1.2%). The average aNDF concentration of the leaves was 48.5% (SEM: 3.0%); it was lowest in La (36.3%) and highest in Pt (62%). The ADF concentration fluctuated between 24.7% (Zi) and 47.7% (Gu), with an average of 33.8% (SEM: 2.5%). The highest TEP concentrations (Table 2) were observed in Gu (16.7%) and La (12.9%), while Pt had the lowest TEP content (3.4%). Fi and Zi had the highest CT concentration (both 6.9%) while the lowest CT concentration (0.7%) was obtained for Kh.

**Table 2:** Proximate composition and secondary plant compounds (% DM) of leaves of the woody plant species used in the Hohenheim gas test.

Species (abbreviation)	DM	OM	CP	aNDF	ADF	TEP	CT
*A. indica* (Az)	95.3	90.8	17.2	36.3	30.9	5.0	3.4
*B. costatum* (Bo)	95.2	90.6	14.8	48.6	33.2	5.9	1.9
*F. sycomorus* (Fi)	95.1	79.2	8.3	40.8	33.1	4.0	6.9
*G. senegalensis* (Gu)	95.8	96.1	11.0	55.5	47.7	12.9	4.4
*K. senegalensis* (Kh)	96.0	93.7	16.2	56.6	27.4	4.0	0.7
*L. microcarpa* (La)	94.3	91.9	9.3	40.3	31.3	16.7	4.2
*P. erinaceus* (Pt)	95.4	92.6	16.6	62.0	42.4	3.4	1.8
*Z. mauritiana* (Zi)	95.4	91.0	10.9	47.8	24.7	4.7	6.9
Mean	95.3	90.7	13.0	48.5	33.8	7.1	3.8
SEM	0.2	1.7	1.2	3.0	2.5	1.6	0.8

Abbreviations: ADF, Acid detergent fibre; aNDF, Amylase‐free neutral detergent fibre; CP, Crude protein; CT, Condensed tannins; DM, Dry matter of air‐dry material; OM, Organic matter; SEM, Standard error of the mean; TEP, Total extractable phenols.

**Table 3:** Proximate composition and secondary plant compounds (% DM) of leaves of the woody plant species used in the preference test.

Species (abbreviation)	DM	OM	CP	aNDF	ADF	TEP	CT
*A. indica* (Az)	92.8	89.5	16.5	36.4	26.2	1.8	0.4
*B. costatum* (Bo)	92.2	90.5	16.0	42.5	23.1	4.9	1.5
*F. sycomorus* (Fi)	91.5	75.7	9.4	36.8	25.9	6.0	4.2
*G. senegalensis* (Gu)	93.1	96.1	9.7	59.3	47.0	9.6	3.7
*K. senegalensis* (Kh)	93.2	92.2	10.4	45.6	34.2	12.6	8.2
*L. microcarpa* (La)	91.7	91.7	9.4	31.4	25.9	12.1	2.5
*P. erinaceus* (Pt)	92.9	92.0	17.1	55.4	32.0	1.7	0.1
*Z. mauritiana* (Zi)	92.8	90.8	11.7	37.9	25.4	10.6	7.7
Mean	92.5	89.8	12.5	43.2	30.0	7.4	3.5
SEM	0.2	2.0	1.1	3.2	2.6	1.5	1.0

Abbreviations: ADF, Acid detergent fibre; aNDF, Amylase‐free neutral detergent fibre; CP, Crude protein; CT, Condensed tannins; DM, Dry matter of air‐dry material; OM, Organic matter; SEM, Standard error of the mean; TEP, Total extractable phenols.

Overall, the mean concentrations of nutrients and secondary plant compounds (all in DM) of the leaves used in the preference test (Table 3) were similar to the mean values of the leaves incubated in the Hohenheim gas test. Again, the CP concentration was lowest in Fi and La (both 9.4%), while the highest CP concentration was determined for Pt (17.1%). Concentrations of aNDF and ADF ranged from 31.4% (La) to 59.3% (Gu) and from 23.1% (Bo) to 47.0% (Gu) in the preference test. Kh and Zi had the highest TEP (> 10%) and CT (> 7%) concentrations. The lowest CT concentrations (< 1%) were measured in Pt and Az (Table 3).

### 3.2 In Vitro Digestibility, Total Gas, and Methane Production of Leaves

The results of 24‐h IVOMD, TGP, and CH_4_ are presented in Table 4. Without PEG, the highest IVOMD was observed in Az (60.6%) and Bo (59.6%), indicating that these leaves were more degradable than the other studied leaves. Consequently, they produced higher volumes of gas, namely 33.6 and 34.0 mL/200 mg incubated DM, respectively. On the other hand, La and Gu yielded the lowest IVOMD (32.3% and 35.6%, respectively) and TGP (7.1 and 9.0 mL/200 mg incubated DM). The lowest CH_4_ concentration (3.7% of total gas) was determined for Gu (Table 4), which was significantly different from Az, Bo, and Kh (14.9%, 15.8%, and 14.6% of total gas, respectively).

**Table 4:** In vitro digestibility of organic matter (IVOMD, %), total gas production (TGP) in 24 h (mL/200 mg incubated DM) and methane concentration of total gas (CH_4_, as % of TGP) of leaves incubated without and with the addition of polyethylene glycol (PEG); mean ± standard deviation (SD).

Species (abbreviation)	No addition of PEG			With addition of PEG		
	IVOMD	TGP	CH_4_	IVOMD	TGP	CH_4_
*A. indica* (Az)	60.6 ± 3.7^a^	33.6 ± 4.4^a^	14.9 ± 7.3^a^	64.6 ± 1.7^a^	38.4 ± 2.1^a^	17.0 ± 8.8^a^
*B. costatum* (Bo)	59.6 ± 0.4^a^	34.0 ± 0.5^a^	15.8 ± 0.4^a^	60.5 ± 0.9^bc^	35.0 ± 1.1^b^	17.2 ± 0.7^a^
*F. sycomorus* (Fi)	49.8 ± 0.6^b^	17.4 ± 0.7^c^	12.3 ± 0.6^ab^	61.5 ± 1.2^b^	31.2 ± 1.5^c^	15.2 ± 1.3^a^
*G. senegalensis* (Gu)	32.3 ± 0.7^c^	9.0 ± 0.9^d^	3.7 ± 1.9^b^	41.9 ± 0.8^f^	20.4 ± 1.0^e^	10.2 ± 2.8^a^
*K. senegalensis (*Kh*)*	48.2 ± 1.0^b^	22.2 ± 1.2^bc^	14.6 ± 5.6^a^	48.8 ± 0.4^e^	22.9 ± 0.4^d^	14.1 ± 4.0^a^
*L. microcarpa* (La)	35.6 ± 2.1^c^	10.5 ± 2.5^d^	7.1 ± 1.1^ab^	51.4 ± 0.0^d^	29.3 ± 0.0^c^	12.3 ± 0.1^a^
*P. erinaceus* (Pt)	47.3 ± 1.7^b^	20.8 ± 0.4^bc^	12.8 ± 3.3^ab^	47.7 ± 0.9^e^	20.3 ± 1.1^e^	14.1 ± 0.1^a^
*Z. mauritiana* (Zi)	49.0 ± 2.7^b^	24.5 ± 3.2^b^	8.8 ± 1.6^ab^	58.9 ± 0.4^c^	36.2 ± 0.5^ab^	11.9 ± 0.7^a^
Overall mean	47.1	20.9	11.1	54.0	28.9	14.0
SEM	2.2	2.0	1.2	1.8	1.5	0.9
*p*‐value (global test)	***	***	*	***	***	0.58

*Note:* Linear model and Tukey HSD (Honestly Significant Difference) post‐hoc test; means in the same column with different superscript letters after the SD differ at ***p < 0.001, **p < 0.01, *p < 0.05, n.s. *p* > 0.05.

Abbreviation: SEM, Standard error of the mean.

The addition of PEG increased TGP for La (179%), Gu (127%), and Fi (79.6%) compared to the incubation without PEG, but slightly decreased TGP for Pt (−2.4%). In consequence, the highest increase in IVOMD was observed for La (+44.4%), Gu (+22.7%), and Fi (+23.5%), while the addition of PEG had no major effect on IVOMD of Pt and Kh. Similarly, the CH_4_ concentration in total gas increased for Gu (+176.4%), La (+74.4%), and Zi (+35.5%), while it slightly decreased for Kh (−3.7%). Overall, PEG addition did not introduce significant differences (*p* > 0.05) between plant species in total gas CH_4_ concentration (Table 4).

In the absence of PEG, IVOMD, TGP and CH_4_ were negatively correlated with TEP (Kendall correlations: r = −0.8; r = −0.7, and r = −0.6; *p* < 0.001). In contrast, the correlation of CT with IVOMD and TGP was nonsignificant (r = −0.2 and r = −0.3; *p* > 0.05), but significant for CH_4_ (r = −0.5; *p* < 0.05).

### 3.3 Preference Test

**Figure 1:**
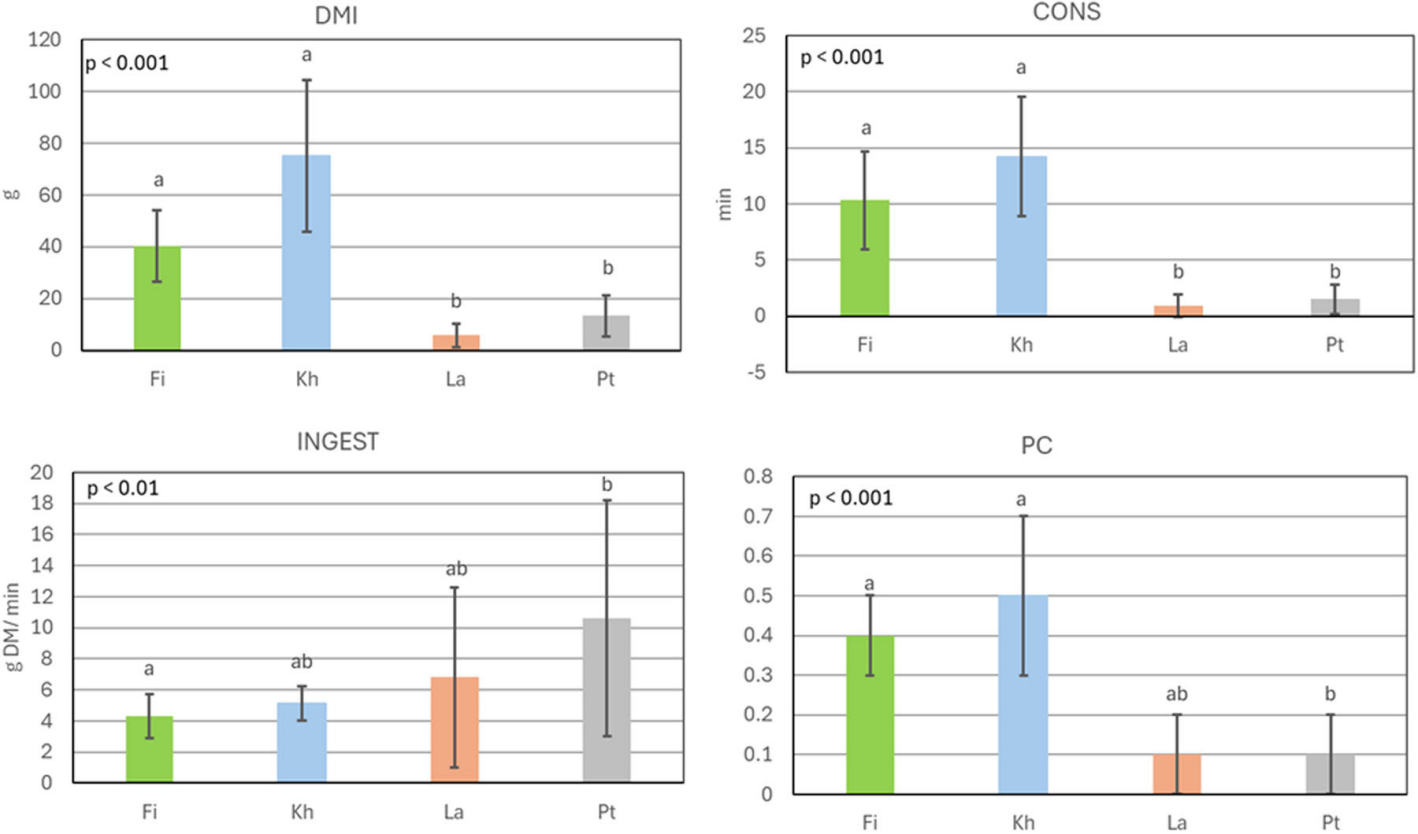
Quantity of consumed dry matter (DMI; g DM), consumption time (CONS; min), ingestion rate (INGEST; g DM/min) and preference coefficient (PC) of four sheep for fresh leaves of *Ficus sycomorus* (Fi), *Khaya senegalensis* (Kh), *Lannea microcarpa* (La) and *Pterocarpus erinaceus* (Pt) during 30 min of observation on 8 days (*n* = 8 observations for each woody plant); mean ± standard deviation. Kruskal‐Wallis and Wilcoxon post hoc tests; means with different superscript letters are different at *p* < 0.05. P‐values in the graphs depict the significance level of the overall model. All x‐axes intersect the y‐axes at zero. [Color figure can be viewed at wileyonlinelibrary.com]

Sheep's preferences for fresh leaves significantly differed between the woody species in both groups (all *p* < 0.05). In group 1 (Figure 1), overall, DMI, CONS, INGEST, and PC of fresh leaves averaged (±SEM) 33.8 ± 2.8 g DM, 6.8 ± 0.6 min, 6.7 ± 0.5 g DM/min, and 0.3 ± 0.0. DMI, CONS, and PC were highest for Kh (75 ± 29.2 g DM; 14 ± 5.3 min; 0.5 ± 0.2) and Fi (40 ± 13.8 g DM; 10 ± 4.4 min; 0.4 ± 0.1), with significant differences from La and Pt (*p* < 0.05). INGEST was highest for Pt (11 ± 7.6 g DM/min) and significantly different from Fi which had the lowest INGEST (4 ± 1.4 g DM/min).

**Figure 2:**
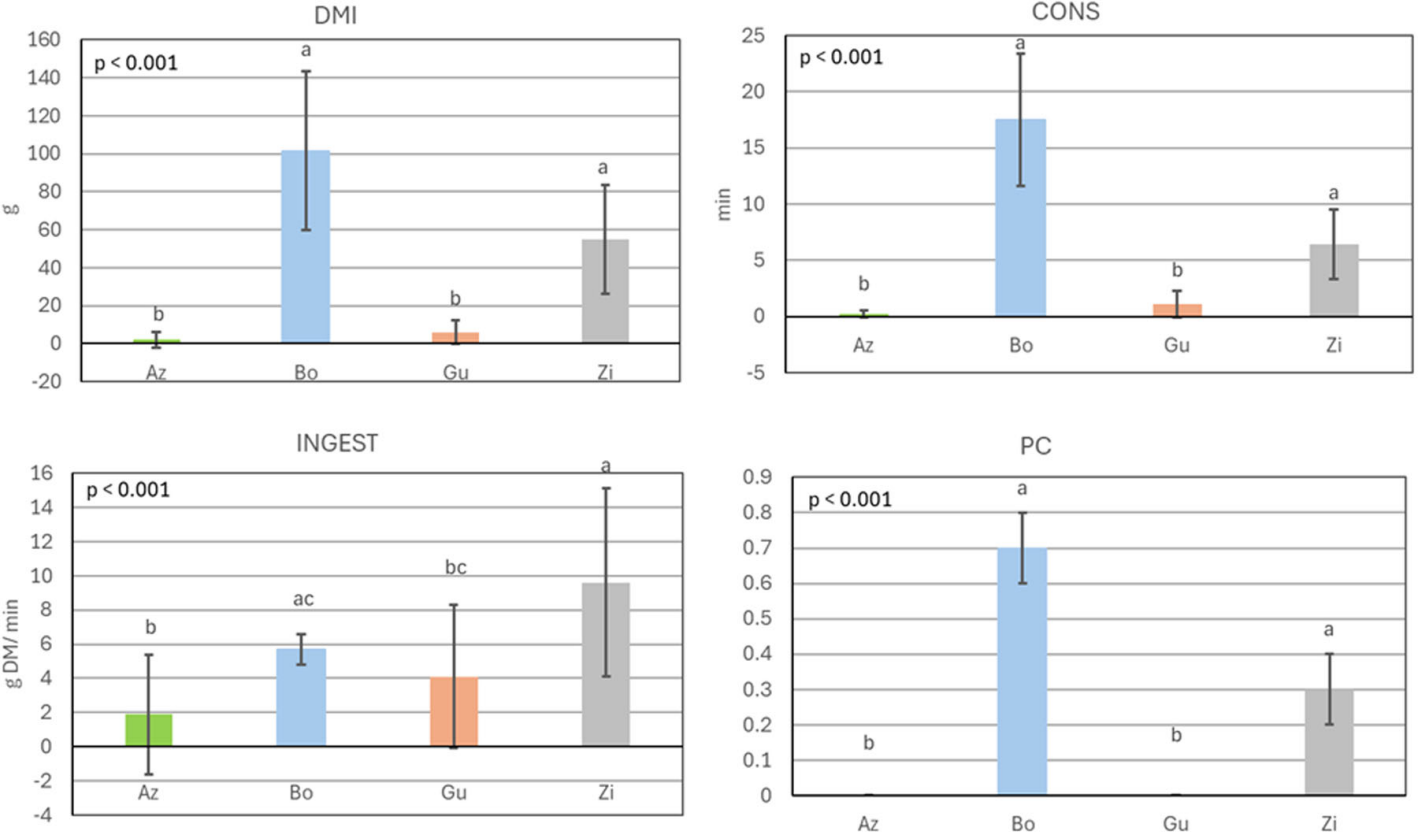
Quantity of consumed dry matter (DMI; g DM), consumption time (CONS; min), ingestion rate (INGEST; g DM/min) and preference coefficient (PC) of four sheep for fresh leaves of *Azadirachta indica* (Az), *Bombax costatum* (Bo), *Guiera senegalensis* (Gu) and *Ziziphus mauritiana* (Zi) during 30 min of observation on 8 days (*n* = 8 observations for each woody plant); mean ± standard deviation. Kruskal‐Wallis and Wilcoxon post hoc tests; means with different superscript letters are different at *p* < 0.05. P‐values in the graphs depict the significance level of the overall model. All x‐axes intersect the y‐axes at zero. [Color figure can be viewed at wileyonlinelibrary.com]

In group 2 (Figure 2), the overall means (±SEM) of DMI, CONS, INGEST, and PC of fresh leaves were 41 ± 4.2 g DM, 6 ± 0.7 min, 5 ± 0.4 g DM/min, and 0.3 ± 0.0, respectively. Bo and Zi had a significantly higher DMI, CONS, and PC than Az and Gu, namely 102 ± 41.8 g DM, 18 ± 5.9 min, 0.7 ± 0.1 (Bo), and 55 ± 28.7 g DM, 6 ± 3.1 min, and 0.3 ± 0.1 (Zi). Az and Gu were the least consumed and preferred. Highest INGEST was observed for fresh leaves of Zi (9.6 ± 5.5 g DM/min), being significantly different from Az and Gu (*p* < 0.05).

**Figure 3:**
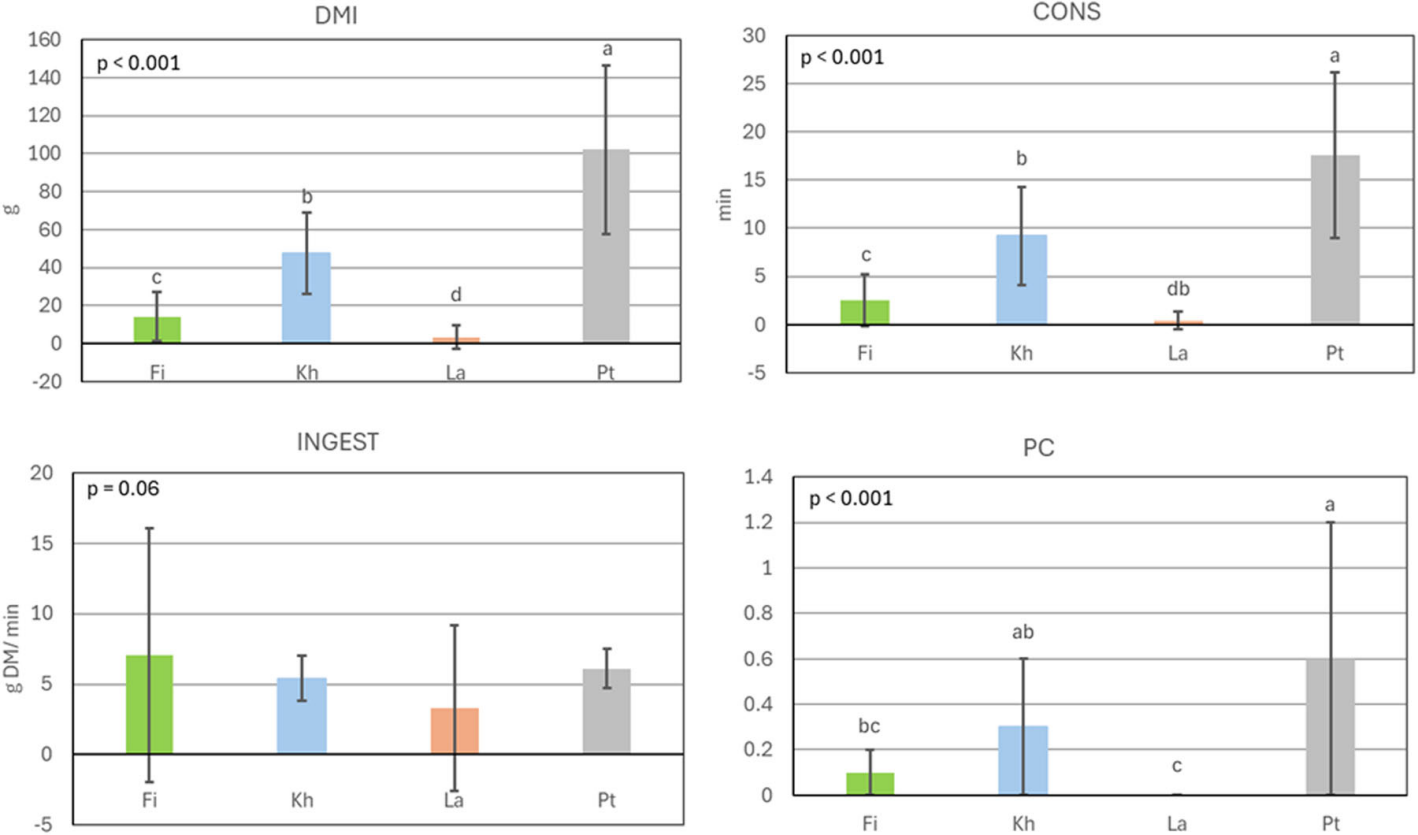
Quantity of consumed dry matter (DMI; g DM), consumption time (CONS; min), ingestion rate (INGEST; g DM/min) and preference coefficient (PC) of four sheep for dried leaves of *Ficus sycomorus* (Fi), *Khaya senegalensis* (Kh), *Lannea microcarpa* (La) and *Pterocarpus erinaceus* (Pt) during 30 min of observation on 8 days (*n* = 8 observations for each woody plant); mean ± standard deviation. Kruskal‐Wallis and Wilcoxon post hoc tests; means with different superscript letters are different at *p* < 0.05. P‐values in the graphs depict the significance level of the overall model. All x‐axes intersect the y‐axes at zero. [Color figure can be viewed at wileyonlinelibrary.com]

**Figure 4:**
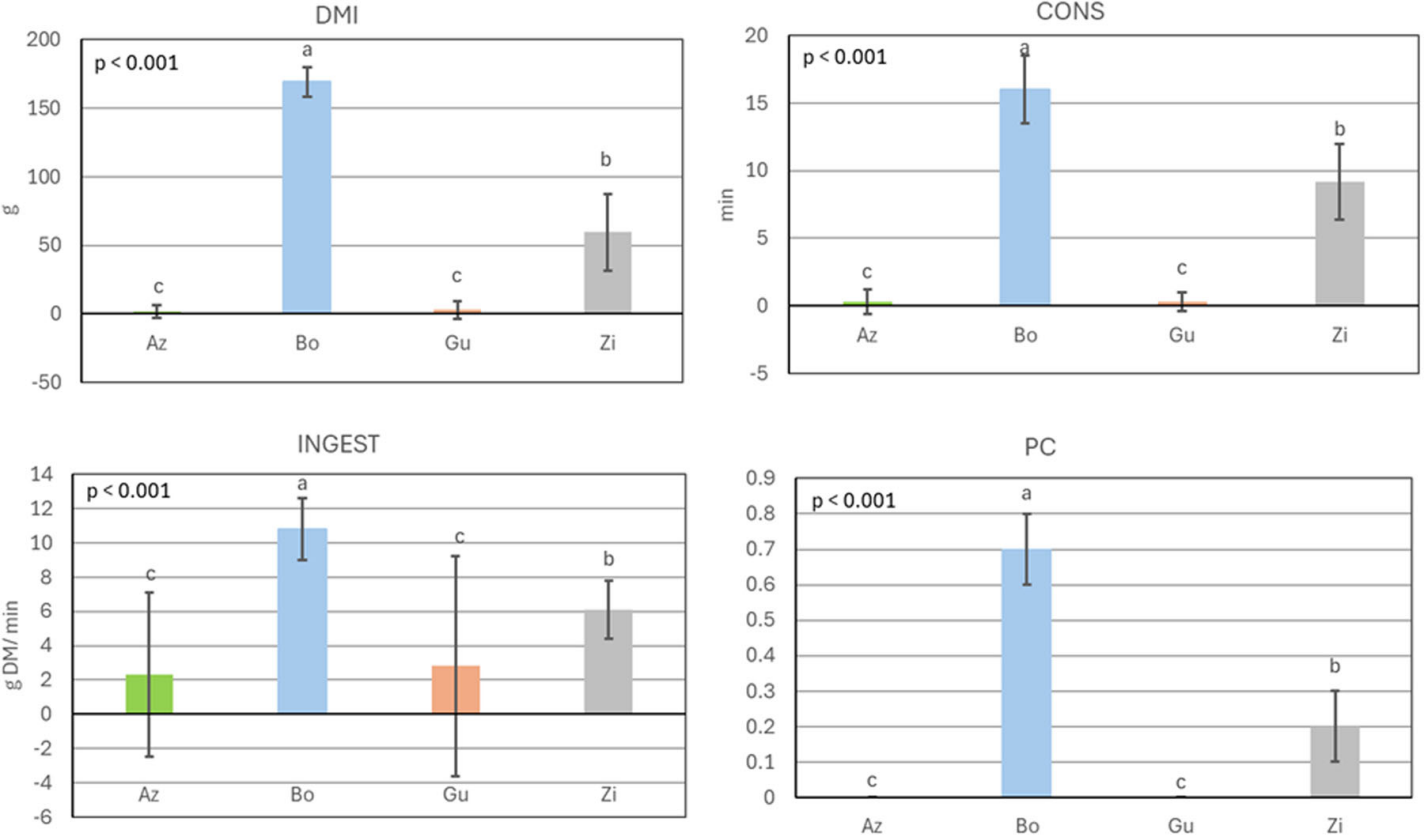
Quantity of consumed dry matter (DMI; g DM), consumption time (CONS; min), ingestion rate (INGEST; g DM/min) and preference coefficient (PC) of four sheep for dried leaves of *Azadirachta indica* (Az), *Bombax costatum* (Bo), *Guiera senegalensis* (Gu) and *Ziziphus mauritiana* (Zi) during 30 min of observation on 8 days (*n* = 8 observations for each woody plant); mean ± standard deviation. Kruskal‐Wallis and Wilcoxon post hoc tests; means with different superscript letters are different at *p* < 0.05. P‐values in the graphs depict the significance level of the overall model. All x‐axes intersect the y‐axes at zero. [Color figure can be viewed at wileyonlinelibrary.com]

The group averages (±SEM) for DMI, CONS, INGEST, and PC of dried leaves in group 1 (Figure 3) were 43 ± 4.1 g DM, 7 ± 0.8 min, 5 ± 0.5 g DM/min, and 0.2 ± 0.0, respectively. Similar to the results for fresh leaves, sheep's preferences for dried leaves significantly differed between the species in both groups (all *p* < 0.05). However, while the results for dried leaves of the species in group 2 (Figure 4) were comparable to those of the fresh leaves, sheep's preferences for dry leaves of the species in group 1 (Figure 3) deviated from those for fresh leaves. Dried leaves of Pt were the most preferred (DMI: 102 ± 44.4 g DM; CONS: 18 ± 8.6 min; PC 0.6 ± 0.2; *p* < 0.05), while dried leaves of La were the least preferred (DMI: 3.4 ± 6.0 g DM; CONS 0.4 ± 0.9 min; PC: 0.0 ± 0.01; *p* < 0.05). There was no significant difference in INGEST between the species in group 1 (Figure 3).

In group 2 (Figure 4), DMI, CONS, INGEST, and PC of dried leaves averaged (±SEM) 59 ± 6.2 g DM, 6 ± 0.6 min, 5 ± 0.5 g DM/min, and 0.3 ± 0.0. The highest preference was recorded for Bo with a DMI of 169 ± 10.6 g DM, 16 ± 2.5 min of CONS, 11 ± 1.8 g DM/min of INGEST and a PC of 0.7 ± 0.1 (*p* < 0.05), followed by Zi with a DMI of 59 ± 28.1 g DM, 9 ± 2.8 min of CONS, 6 ± 1.7 g DM/min of INGEST and a PC of 0.2 ± 0.1 (*p* < 0.05). Az and Gu were the least preferred species when offered dry.

Table 5 presents the results of the Pearson correlation between the proximate and secondary plant compounds and DMI as well as CONS. DMI and CONS of fresh leaves of species in group 1 were positively and significantly correlated with the concentration of DM, TEP, CT, aNDF, and ADF (r ≥ 0.4; *p* < 0.001). In contrast, DMI and CONS in group 2 showed a significant negative correlation with DM, aNDF, and ADF (r = −0.8, −0.5, −0.6 and r = −0.8, −0.4, −0.6, respectively; all *p* < 0.001). However, no association was observed between DMI or CONS and the secondary plant compounds (TEP, CT) of the leaves (*p* > 0.05). In both groups, DMI and CONS of fresh leaves were also not influenced by CP concentration (Table 5).

**Table 5:** Significance levels* of Pearson correlations between dry matter intake (DMI, g DM in 30 min) and consumption time (CONS, minutes) of fresh and dried leaves of two groups of four woody species each, and specific chemical and secondary plant compounds** (*n* = 64 observations per tested correlation).

	Fresh leaves				Dried leaves				
Variable	DMI	*p*‐value	CONS	*p*‐value	Group 1	DMI	*p*‐value	CONS	*p*‐value
Group 1									
DM	0.6	***	0.5	***	DM	0.6	***	0.6	***
TEP	0.4	***	0.4	**	TEP	−0.6	***	−0.5	***
TC	0.8	***	0.8	***	TC	−0.3	*	−0.2	n.s.
CP	−0.1	n.s.	−0.2	n.s.	CP	0.8	***	0.7	***
aNDF	0.4	***	0.2	n.s.	aNDF	0.1	n.s.	0	n.s.
ADF	0.5	***	0.4	***	ADF	0.6	***	0.6	***
Group 2									
DM	−0.8	***	−0.8	***	DM	−0.9	***	−0.84	***
TEP	−0.1	n.s.	−0.2	n.s.	TEP	−0.1	n.s.	0.06	n.s.
TC	0.04	n.s.	−0.1	n.s.	TC	−0.1	n.s.	0.14	n.s.
CP	−0.1	n.s.	−0.1	n.s.	CP	0.4	***	0.3	*
aNDF	−0.5	***	−0.4	***	aNDF	−0.3	n.s.	−0.3	**
ADF	−0.6	***	−0.6	***	ADF	−0.6	***	−0.6	***

*Note:* Significance levels of Pearson correlations: ***p < 0.001, **p < 0.01, *p < 0.05, n.s. *p* > 0.05. Concentrations of chemical and secondary plant compounds for fresh leaves in g/kg fresh matter, for dried leaves in g/kg DM. Group 1: *Lannea microcarpa*, *Ficus sycomorus*, *Pterocarpus erinaceus*, *Khaya senegalensis*; Group 2: *Azadirachta indica*, *Bombax costatum*, *Guiera senegalensis*, *Ziziphus mauritiana*.

Abbreviations: ADF, Acid detergent fibre; aNDF, Amylase‐free neutral detergent fibre; CP, Crude protein; CT, Condensed tannins; DM, Dry matter; TEP, Total extractable phenols.

For dried leaves of group 1, there was a positive and significant correlation between DMI and CONS with DM, CP, and ADF, respectively (r ≥ 0.3; *p* < 0.05), and a negative and significant correlation with TEP (r = −0.6 and −0.5; *p* < 0.05). In group 2, like for the fresh leaves, there was a negative and significant correlation between CONS and DM, aNDF, and ADF (r = −0.8; r = −0.3; r = −0.6; *p* < 0.05), whereas DMI was not significantly correlated with aNDF. CP showed a significant positive correlation with both DMI and CONS in the dried leaves of group 2 (Table 5). In contrast, the correlations of TEP and CT with DMI and CONS were not significant (*p* > 0.05).

Table 6 presents the Pearson correlation test results between PC and IVDOM. A significant positive correlation was observed for fresh leaves in group 1 (r = 0.61, *p* < 0.001) and for dry leaves in both group 1 (r = 0.61, *p* < 0.001) and group 2 (r = 0.40, *p* = 0.001).

**Table 6:** Pearson correlations between preference coefficient and in vitro digestibility of organic matter (IVOMD) for different groups of fresh and dried leaves.

	Fresh leaves		Dried leaves	
Variable	IVOMD	*p*‐value	IVOMD	*p*‐value
Group 1				
Preference coefficient	0.61	*	0.39	*
Group 2				
Preference coefficient	0.24	n.s.	0.40	*

*Note:* Significance levels of Pearson correlations: *p < 0.001, n.s. *p* > 0.05. Group 1: *Lannea microcarpa, Ficus sycomorus, Pterocarpus erinaceus, Khaya senegalensis*; Group 2: *Azadirachta indica*, *Bombax costatum*, *Guiera senegalensis*, Ziziphus mauritiana.

## 4 Discussion

### 4.1 Chemical Composition of Selected Woody Species

For the woody species studied, the chemical composition varied between the leaf samples subjected to the in vitro digestibility test (harvested in the dry season) and the preference test (harvested in the rainy season), probably due to the stage of leaf development during the harvesting period. During the rainy season, all studied species bear fresh leaves, while at the end of the dry season, the leaves mature and fall off. Leaf ageing is negatively correlated with the concentration of all chemical compounds except ADL (Mboko et al. 2017; Ouédraogo et al. 2021). Hence, it would be worthwhile for farmers to harvest, dry, and store leaves before they reach senescence. Despite seasonal differences, all leaf samples in our study had a CP content (in DM) of 9%–17% (preference test) and 8%–17% (in vitro digestibility test), which is higher than the 7%–8% required for proper rumen function (Van Soest 1994). Our values exceed those of Mebirouk‐Boudechiche et al. (2015) in Algeria (1.1%–13.9%, *Viburnum tinus, Acacia dealbata*) and are comparable to those reported by Dione et al. (2022) for Senegal (7.8%–18.2%, *Eucalyptus alba, Piliostigma reticulatum*). The species tested are therefore well suited for CP supplementation of ruminant diets at the end of the dry season, when feed resources are scarce and of poor quality. The concentrations (in DM) of aNDF of 31%–59% (preference test) and 36%–55% (in vitro digestibility test) were lower than those reported by Dione et al. (2022) for Senegal (38.5%–67.5%; *Adansonia digitata*, *Heeria insignis*) and Koura et al. (2021) for the Sudano‐Guinean zone of Benin (30.7%–68.7%; *Moringa oleifera, Bambusa vulgaris)*. For ADF (23%–47% in DM, both tests), the values were lower than those reported by Dione et al. (2022) (25.1%–65.5%; *Balanites aegyptiaca, Heeria insignis*) but higher than those determined by Koura et al. (2021) (13.9%–38.4%; *Musa sapientum, Bambusa vulgaris*). According to Mebirouk‐Boudechiche et al. (2015), the high fibre (lignin) content of woody plants may limit their digestibility; however, lignin is mostly concentrated in soft twigs that are harvested with the leaves, not in the leaves themselves. Therefore, differences in cell wall constituents and phytochemicals between studies can also be attributed to variations in leaf harvesting methods (Rubanza et al. 2003). In addition to fibre, a high CT content (6%–12% DM) can reduce intake and digestibility (Okunade et al. 2014). We found that the addition of PEG increased the in vitro digestibility of Fi by 23% compared to Bo, whose digestibility remained almost unchanged. However, the average CT concentration (in DM) of the leaves in our tests was relatively low: 3.5% (preference test) and 3.8% (in vitro digestibility test), ranging from 0.1% (Pt) to 8.2% (Kh) (preference test) and from 0.7 (Kh) to 6.9% (Fi, Zi) (in vitro digestibility test). These results are comparable to those of Koura et al. (2021) in Benin (0%–6.2% CT in DM; *Ficus thonningii, Mangifera indica*) and Okunade et al. (2014) in Nigeria (0.2%–5.9% CT in DM; *Securnega virosa, Afzelia africana*).

### 4.2 In Vitro Digestibility, Total Gas, and Methane Production of Leaves

The IVOMD of the leaves of the studied browse species ranged from 32.3% for *G. senegalensis* to 59.6% for *B. costatum*, with an average of 47.1%. These values are slightly higher than those reported from Benin (Sidi Imorou et al. 2016), where the digestibility of 26 browse species ranged from 35.1% (*Stereospermum kunthianum*) to 54.8% (*Phyllanthus muellerianus*), with an average of 43.8%. The positive correlations between IVOMD and the preference coefficient of most species indicates that plants highly preferred by the animals were more digestible, or, in other words, that the animals preferred well‐digestible leaves. These results are consistent with those of Kyambu et al. (2021), who showed that the selectivity index increases with higher IVDMD (r = 0.563). According to the principle of the used in vitro method, higher TGP values resulted in increased IVOMD, suggesting enhanced microbial fermentation activity in the rumen fluid used for incubation, an improvement primarily due to easier breakdown of carbohydrate fractions (Blama et al. 2022).

The increase in TGP, and consequently IVOMD, that was observed with PEG addition in leaves with a high CT content, along with the significant negative correlation between CT and both IVOMD and TGP in the absence of PEG, points to the inhibitory effect of CT on microbial fermentation (µ; Mebirouk‐Boudechiche et al. 2015; Nascimento et al. 2021; Battelli et al. 2023). PEG has a strong binding affinity for CT and replaces tannin‐protein complexes with tannin‐PEG complexes (Elahi et al. 2014), thereby eliminating or reducing the inhibitory effect of tannins on in vitro digestibility, as highlighted by several authors (Elahi et al. 2014; Mebirouk‐Boudechiche et al. 2015; Nascimento et al. 2021). PEG addition also led to an increase in CH_4_ production for the leaves of all woody species. Fodder plants with high biologically active CT can therefore help to reduce the emission of enteric CH_4_, an important greenhouse gas, as also described by Mebirouk‐Boudechiche et al. (2015) and Hammami et al. (2023). However, for Kh, there was a decrease in CH_4_ (−3.7%) when adding PEG. The CT content of Kh was very low, which could explain the low or absent biological activity. In their study conducted in Guadeloupe, Archimède et al. (2015) observed that leaves rich in CT (*Glyricidia sepium, Leucaena leucocephala*, and *Manihot esculenta*) had no effect on organic matter digestibility but reduced CH_4_ production of sheep. Beyond concentration, these authors suggested that CT effects also depend on their structural configuration.

### 4.3 Leaves Highly Preferred by Sheep

The aim of this study was to identify woody fodder species that combine animal preference with good nutritional value, in particular digestibility, in the northern Sudanian zone of Burkina Faso, for improved dry season feeding of stabulated animals. Kyambu et al. (2021) stated that the amount of ingested DM is a key criterion for assessing forage preference. According to the preference coefficient based on DMI, Kh in group 1, and Bo and Zi in group 2 were the most preferred species, regardless of leaf condition (fresh or dried). In group 1, Pt was more preferred in the dried state, while Fi leaves were more appreciated in the fresh state. For the latter species, there was a positive correlation between aNDF and ADF content and DMI, for both dried and fresh leaves. These results are in line with those of Hernández‐Orduño et al. (2015) who found a positive correlation between ADF and NDF, respectively, and feed intake, but a negative correlation of the latter variable with lignin content. Okunade et al. (2014) and Ouédraogo et al. (2021) found that high DM, ADF, and NDF concentrations reduced feed intake.

Intake of dry leaves was negatively correlated with their CT content, which could explain why Fi (4.2% CT in DM) was less preferred in the dried state than Pt (0.1% CT in DM). Soulama et al. (2014) noted that dry leaves contain more tannins than fresh ones. Specific physical and olfactory characteristics could add to this by inducing a higher interest of sheep in dried Pt than dried Fi leaves. Therefore, farmers might be advised to combine highly preferred but tannin‐rich browse species (Zi, Fi) with those containing lower tannin levels (Bo, Kh) during the dry season to balance the overall tannin concentration in the offered foliage.

Beyond various benefits and challenges of leaf consumption, Provenza (2006) and Villalba et al. (2015) observed that past feeding experiences influence the preferences of animals, which learn on pasture to select plants that meet their nutritional needs and which, through their secondary compounds, act against certain infections, especially with intestinal parasites.

According to Van Soest (1994) the ideal nutritional characteristics of a forage to cover the basic needs of ruminants are: > 8% CP, < 50% NDF and < 40% ADF in DM. Apart from Gu and Pt, the ligneous plants tested in our study meet these criteria. However, it is important to note that the leaves of woody plants are not the sole source of feed for grazing or stall‐fed small ruminants; rather, they typically constitute only about one‐third of their daily ration (Aruwayo and Adeleke 2019).

In addition to the quantity ingested, the rate of ingestion is also a means of assessing palatability as noted by Baumont (1996) and Hernández‐Orduño et al. (2015). Although we were unable to prove the existence of significant correlations between INGEST and chemical and anti‐nutritional plant compounds, we were able to show that Pt, with the lowest CT content, had the highest INGEST (10.6 ± 7.6 g DM/min) despite its DMI being among the lowest values (13.3 ± 7.9 g DM). The higher INGEST indicates that this species may have physical and chemical attributes that made it rapidly attractive or easy to consume by sheep in our study. The same observation was made by Hernández‐Orduño et al. (2015) in their study where the plants with highest quantitative ingestion had the lowest INGEST, linked to the size and density of their leaves. For these plants, the animals had to spend more time ingesting them to be saturated, as the leaves were more difficult to chew, took longer to swallow, or required more effort to be consumed in large quantities. Despite this, animals preferred these low‐density leaves and took the time needed to eat them.

## 5 Conclusion

The eight woody plant species examined in this study exhibit high CP levels, making them valuable supplements for sheep rations in the northern Sudanian zone of Burkina Faso, particularly at the end of the dry season. Sheep showed a strong preference for leaves of *B. costatum, K. senegalensis, Z. mauritiana*, and *P. erinaceus* when dried, and *F. sycomorus* when fresh. However, *P. erinaceus* is classified as endangered by the IUCN, while *Z. mauritiana* and *F. sycomorus* contain over 6% CT, which may negatively impact the digestibility of the overall diet. Nonetheless, moderate inclusion levels could mitigate this effect. To enhance feed availability, farmers could prioritise harvesting the leaves of *B. costatum* and *K. senegalensis* toward the end of their fruit maturation, then dry and store them for use during the dry season. In this context, sustainable leaf harvesting practices must be followed, and use of natural stands of *P. erinaceus* should be cautioned.

## Author Contributions

**Linda C. Gabriella Traore:** experimentation, data analysis, drafting of manuscript; **Sita Sanou:** planning, supervision, correction of manuscript. **H. Oumou Sanon:** planning, supervision, funding, correction of manuscript. **Regina Roessler:** study design, coordination of laboratory analysis, data analysis, translation and correction of manuscript. **Valérie Bougouma‐Yameogo:** supervision, correction of manuscript. **Eva Schlecht:** study design, funding, correction of manuscript.

## Ethics Statement

The authors confirm that the ethical policies of the journal, as noted on the journal's author guidelines page, have been adhered to and the appropriate ethical review committee approval has been received. Ethical authorisation for this study and animal experimentation was obtained from the Ethics Committee of Joseph Ki‐Zerbo University in Ouagadougou (number CE‐UJKZ/2022‐07).

## Conflicts of Interest

The authors declare no conflicts of interest.

## Supporting information

Cafeteria trial Appendix.

## Data Availability

The data that support the findings of this study are available on request from the corresponding author. The data are not publicly available due to privacy or ethical restrictions.
