# Supplementary material for: Preference and In Vitro Digestibility of Leaves of Woody Plants by Sheep in the Northern Sudanian Zone
Source: J Anim Physiol Anim Nutr (Berl). 2025 Nov 28;110(2):200–12. doi: 10.1111/jpn.70032 (PMC13000971; doi:10.1111/jpn.70032)
Supplement: Supplementary file 1 — Cafeteria trial Appendix. [file JPN-110-200-s001.docx]

**Appendix 1: Experimental setup of the palatability study with fresh and dry leaves**

| **Period 1: Fresh leaves, 650 g fresh matter/ram** | | | |  |  |  |  |
| --- | --- | --- | --- | --- | --- | --- | --- |
| **Period 2: Dry leaves, 200 g dry matter/ram** | | | |  |  |  |  |
|  |  |  |  | **Feeders** | | | |
| Day | Time | Sheep Observer 1 | Sheep Observer 2 | Position 1 | Position 2 | Position 3 | Position 4 |
| 1 | 8:00-8:30 | A | D | Fi | Pt | La | Kh |
| 1 | 8:45-9:15 | B | C | La | Fi | Kh | Pt |
| 2 | 8:00-8:30 | A | D | Az | Bo | Gu | Zi |
| 2 | 8:45-9:15 | B | C | Bo | Az | Zi | Gu |
| 3 | 8:00-8:30 | A | D | Kh | La | Pt | Fi |
| 3 | 8:45-9:15 | B | C | Pt | Kh | Fi | La |
| 4 | 8:00-8:30 | A | D | Zi | Az | Bo | Gu |
| 4 | 8:45-9:15 | B | C | Gu | Zi | Az | Bo |
| 5 | 8:00-8:30 | A | D | La | Fi | Kh | Pt |
| 5 | 8:45-9:15 | B | C | Kh | La | Pt | Fi |
| 6 | 8:00-8:30 | A | D | Bo | Gu | Zi | Az |
| 6 | 8:45-9:15 | B | C | Az | Bo | Gu | Zi |
| 7 | 8:00-8:30 | A | D | Pt | Kh | Fi | La |
| 7 | 8:45-9:15 | B | C | Fi | Pt | La | Kh |
| 8 | 8:00-8:30 | A | D | Gu | Zi | Az | Bo |
| 8 | 8:45-9:15 | B | C | Zi | Gu | Bo | Az |

Tested ligneous species*: Azadirachta indica* (Az), *Bombax costatum* (Bo), *Ficus sycomorus* (Fi), *Guiera senegalensis* (Gu), *Khaya senegalensis* (Kh), *Lannea microcarpa* (La), *Pterocarpus erinaceus* (Pt), *Ziziphus mauritiana* (Zi).
